# Long-Term Care Workers in South Korea and Their Protection of Human Rights of Persons Living With Dementia

**DOI:** 10.1093/geroni/igaa057.1761

**Published:** 2020-12-16

**Authors:** Hyun Ji Lee, Kyong Hee Chee

**Affiliations:** 1 Daegu Catholic University, Daegu, Republic of Korea; 2 Texas State University, San Marcos, Texas, United States

## Abstract

South Korea has included dementia care as part of their Long-Term Care (LTC) Insurance Program since 2014. Thus, LTC workers are increasingly engaged in dementia care. The purpose of this paper is to elucidate the role of the LTC workers’ occupational identity in their consciousness of human rights as well as their practice of human rights protection. Data were collected in 2018 from 303 LTC workers employed by 12 LTC facilities who completed questionnaires. Their mean age was 53.6 years. We measured occupational identity with a 15-item scale (a=.772), consciousness of human rights for persons living with dementia (PLWD) with a 27-item scale (a=.916), and practice of human rights for PLWD with a 26-item scale (a=.956). Results show that occupational identity was positively associated with consciousness of human rights (r=.296, p<.01) and practice of human rights (r=.295, p<.01). The findings have implications for protecting the human rights of PLWD. Part of a symposium sponsored by the Aging Among Asians Interest Group.

